# 9-(4-Hy­droxy-3-meth­oxy­phen­yl)-3,3,6,6-tetra­methyl-1,2,3,4,5,6,7,8,9,10-deca­hydro­acridine-1,8-dione

**DOI:** 10.1107/S1600536812050568

**Published:** 2012-12-15

**Authors:** Rajni Kant, Vivek K. Gupta, Kamini Kapoor, D. R. Patil, P. P. Patil, Madhukar B. Deshmukh

**Affiliations:** a X-ray Crystallography Laboratory, Post-Graduate Department of Physics & Electronics, University of Jammu, Jammu Tawi 180 006, India; b Department of Chemistry, Shivaji University, Kolhapur 416 004, India; c Department of Agrochemicals and Pest Management, Shivaji University, Kolhapur 416 004 (MS), India.

## Abstract

In the title mol­ecule, C_24_H_29_NO_4_, the central ring of the acridinedione system adopts a flat boat conformation and the four essentially planar atoms of this ring [maximum deviation = 0.001 (2) Å] form a dihedral angle of 85.99 (12)° with the benzene ring. The two outer rings of the acridinedione system adopt sofa conformations. In the crystal, O—H⋯O and N—H⋯O hydrogen bonds link the mol­ecules, forming a two-dimensional network parallel to (100).

## Related literature

For applications of acridines, see: Murugan *et al.* (1998 ▶); Josephrajan *et al.* (2005 ▶); Srividya *et al.* (1998 ▶,1996 ▶). For related structures, see: Balamurugan *et al.* (2009 ▶); Zhao & Teng (2008 ▶). For ring conformations, see: Duax & Norton (1975 ▶).
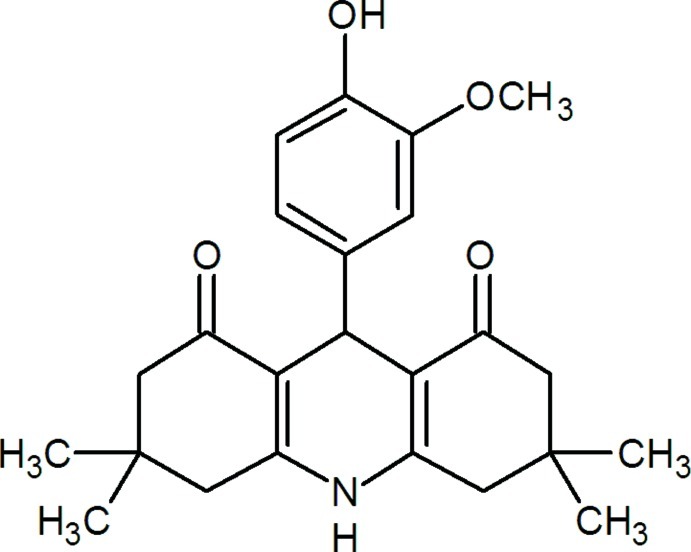


## Experimental

### 

#### Crystal data

C_24_H_29_NO_4_
*M* *_r_* = 395.48
Monoclinic, 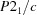

*a* = 10.4828 (3) Å
*b* = 14.8973 (4) Å
*c* = 14.2059 (3) Å
β = 101.609 (2)°
*V* = 2173.09 (10) Å^3^
*Z* = 4
Mo *K*α radiation
μ = 0.08 mm^−1^
*T* = 293 K
0.3 × 0.2 × 0.2 mm

#### Data collection

Oxford Diffraction Xcalibur Sapphire3 diffractometer
Absorption correction: multi-scan (*CrysAlis PRO*; Oxford Diffraction, 2010 ▶) *T*_min_ = 0.970, *T*_max_ = 1.000
63634 measured reflections
4264 independent reflections
2958 reflections with *I* > 2σ(*I*)
*R*_int_ = 0.078

#### Refinement

*R*[*F* ^2^ > 2σ(*F* ^2^)] = 0.054
*wR*(*F* ^2^) = 0.116
*S* = 1.02
4264 reflections
267 parameters
H-atom parameters constrained
Δρ_max_ = 0.19 e Å^−3^
Δρ_min_ = −0.17 e Å^−3^

### 

Data collection: *CrysAlis PRO* (Oxford Diffraction, 2010 ▶); cell refinement: *CrysAlis PRO*; data reduction: *CrysAlis PRO*; program(s) used to solve structure: *SHELXS97* (Sheldrick, 2008 ▶); program(s) used to refine structure: *SHELXL97* (Sheldrick, 2008 ▶); molecular graphics: *PLATON* (Spek, 2009 ▶); software used to prepare material for publication: *PLATON*.

## Supplementary Material

Crystal structure: contains datablock(s) I, global. DOI: 10.1107/S1600536812050568/lh5567sup1.cif

Structure factors: contains datablock(s) I. DOI: 10.1107/S1600536812050568/lh5567Isup2.hkl

Supplementary material file. DOI: 10.1107/S1600536812050568/lh5567Isup3.cml

Additional supplementary materials:  crystallographic information; 3D view; checkCIF report

## Figures and Tables

**Table 1:** Hydrogen-bond geometry (Å, °)

*D*—H⋯*A*	*D*—H	H⋯*A*	*D*⋯*A*	*D*—H⋯*A*
O1—H1⋯O4^i^	0.82	2.12	2.800 (2)	141
N10—H10⋯O3^ii^	0.86	1.95	2.802 (2)	174

Symmetry codes: (i) 
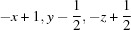
; (ii) 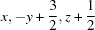.

**Fig. 1.**
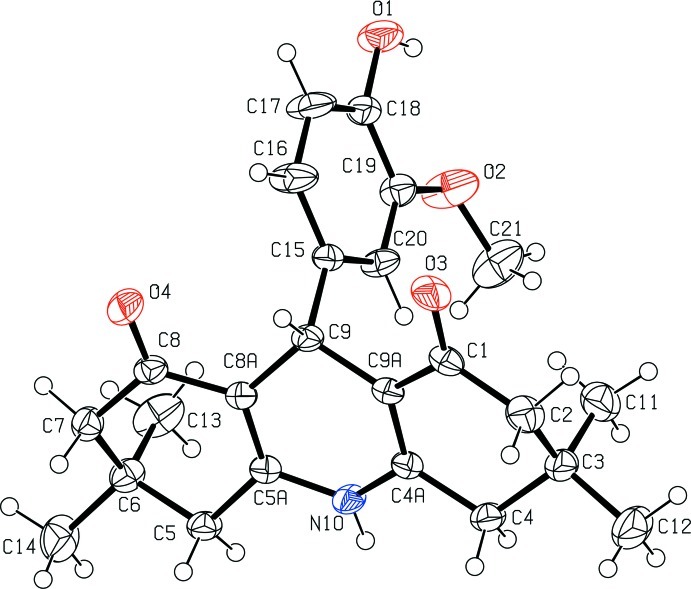
The molecular structure of the title compound with ellipsoids drawn at the 40% probability level. H atoms are shown as small spheres of arbitrary radii.

**Fig. 2.**
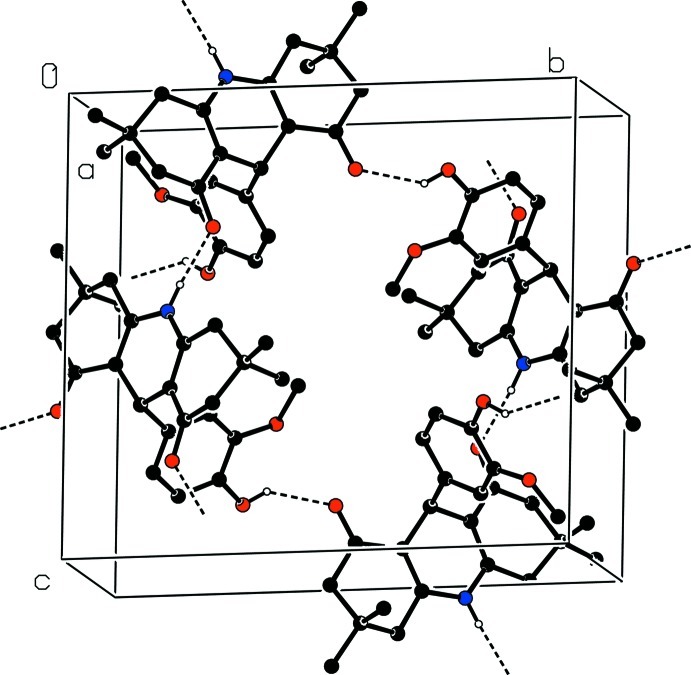
The packing arrangement of molecules viewed along the a axis. The dashed lines show intermolecular O—H···O and N—H···O hydrogen bonds.

## supplementary crystallographic information

### Comment

Acridines, the earliest known antibiotics, are toxic towards bacteria. Some acridinedione derivatives show good inhibition against the pathogen Vibrio isolate-I (Josephrajan *et al.*, 2005). Certain acridine-1,8-diones exhibit fluorescence activities (Murugan *et al.*, 1998) and a few acridinedione derivatives also show photophysical (Srividya *et al.*, 1998) and electrochemical properties (Srividya *et al.*, 1996). Thus, the accurate description of crystal structures of substituted acridinediones are expected to provide useful information on the role of substituents in influencing molecular conformation which has a direct relationship to biological activity. This paper deals with the crystal structure of a 4-hydroxy-3-methoxyphenyl substituted tetramethyl acridinedione, (I).

In (I) (Fig.1), all bond lengths and angles are normal and correspond to those observed in related structures (Balamurugan *et al.*,2009; Zhao & Teng 2008). The central ring (C4A/C5A/C8A/C9A/C9/N10) of the acridinedione moiety adopts a boat conformation (ΔCs(N10) = 0.129 & ΔCs (C5A—C8A) = 10.84) and the four essentially planar atoms (C4A/C5A/C8A/C9A) of this ring (maximum deviation 0.001 (2)Å for all atoms) forms a dihedral angle of 85.99 (12)° with benzene ring. Both the outer rings adopt sofa conformations (ΔCs (C6) = 1.55; ΔCs (C3) = 7.45) (Duax & Norton, 1975). In the crystal, O1—H1···O4^i^ and N10—H10···O3^ii^ hydrogen bonds (Table 1) link molecules to form a two-dimensional network parallel to (100) (Fig. 2).

### Experimental

In a 50 ml rounded bottom flask, a mixture of dimedone (2 mmole), 4-hydroxy, 3-methoxy benzaldehyde (1 mmole) and ammonium acetate (1.2 mmole) in mixture of aqueous ethanol (7 ml) was stirred at RT for 5 min. To this mixture 3-carboxymethyl-1-methylimidazolium(HSO_4_) (20 mol%) was added and the reaction mixture heated at 348-353K for 1.5 hrs. The progress of reaction was monitored by TLC. After completion of reaction, the mixture was cooled to RT and poured on iced water under stirring, The precipitate was filter and dried. The crude product were recrystallized from ethanol to afford X-ray quality crystals. M.P.: 568–571 K, Yield: 82%. IR(KBr): 3274,3168,3049,1623,1511,1370 cm-1. 1H NMR(300 MHz, DMSO-d6): δ = 8.7(s,1*H*,OH);7.7 (s, 1H, NH); 6.7 (s, 1H, Ar—H); 6.5(s,2*H*,Ar—H); 4.7 (s, 1H, CH); 3.67 (s, 3H, OCH3); 2.4–2.0 (m, 8H, CH2); 1.0 (s, 6H, CH3); 0.9 (s,6*H*, CH3).

### Refinement

All H atoms were positioned geometrically and were treated as riding on their parent C atoms, with C—H distances of 0.93–0.96 Å and with *U*_iso_(H) = 1.2*U*_eq_(C) or 1.5*U*_eq_(methyl C).

### Crystal data

**Table:**

C_24_H_29_NO_4_	*F*(000) = 848
*M**_r_* = 395.48	*D*_x_ = 1.209 Mg m^−^^3^
Monoclinic, *P*2_1_/*c*	Mo *K*α radiation, λ = 0.71073 Å
Hall symbol: -P 2ybc	Cell parameters from 22356 reflections
*a* = 10.4828 (3) Å	θ = 3.5–29.2°
*b* = 14.8973 (4) Å	µ = 0.08 mm^−^^1^
*c* = 14.2059 (3) Å	*T* = 293 K
β = 101.609 (2)°	Block, yellow
*V* = 2173.09 (10) Å^3^	0.3 × 0.2 × 0.2 mm
*Z* = 4	

### Data collection

**Table:**

Oxford Diffraction Xcalibur Sapphire3 diffractometer	4264 independent reflections
Radiation source: fine-focus sealed tube	2958 reflections with *I* > 2σ(*I*)
Graphite monochromator	*R*_int_ = 0.078
Detector resolution: 16.1049 pixels mm^-1^	θ_max_ = 26.0°, θ_min_ = 3.5°
ω scans	*h* = −12→12
Absorption correction: multi-scan (*CrysAlis PRO*; Oxford Diffraction, 2010)	*k* = −18→18
*T*_min_ = 0.970, *T*_max_ = 1.000	*l* = −17→17
63634 measured reflections	

### Refinement

**Table:**

Refinement on *F*^2^	Primary atom site location: structure-invariant direct methods
Least-squares matrix: full	Secondary atom site location: difference Fourier map
*R*[*F*^2^ > 2σ(*F*^2^)] = 0.054	Hydrogen site location: inferred from neighbouring sites
*wR*(*F*^2^) = 0.116	H-atom parameters constrained
*S* = 1.02	*w* = 1/[σ^2^(*F*_o_^2^) + (0.0342*P*)^2^ + 1.3025*P*] where *P* = (*F*_o_^2^ + 2*F*_c_^2^)/3
4264 reflections	(Δ/σ)_max_ = 0.001
267 parameters	Δρ_max_ = 0.19 e Å^−^^3^
0 restraints	Δρ_min_ = −0.17 e Å^−^^3^

### Special details

**Table:**

Experimental. *CrysAlis PRO*, Oxford Diffraction Ltd., Version 1.171.34.40 (release 27–08-2010 CrysAlis171. NET) (compiled Aug 27 2010,11:50:40) Empirical absorption correction using spherical harmonics, implemented in SCALE3 ABSPACK scaling algorithm.
Geometry. All e.s.d.'s (except the e.s.d. in the dihedral angle between two l.s. planes) are estimated using the full covariance matrix. The cell e.s.d.'s are taken into account individually in the estimation of e.s.d.'s in distances, angles and torsion angles; correlations between e.s.d.'s in cell parameters are only used when they are defined by crystal symmetry. An approximate (isotropic) treatment of cell e.s.d.'s is used for estimating e.s.d.'s involving l.s. planes.
Refinement. Refinement of *F*^2^ against ALL reflections. The weighted *R*-factor *wR* and goodness of fit *S* are based on *F*^2^, conventional *R*-factors *R* are based on *F*, with *F* set to zero for negative *F*^2^. The threshold expression of *F*^2^ > σ(*F*^2^) is used only for calculating *R*-factors(gt) *etc*. and is not relevant to the choice of reflections for refinement. *R*-factors based on *F*^2^ are statistically about twice as large as those based on *F*, and *R*- factors based on ALL data will be even larger.

### Fractional atomic coordinates and isotropic or equivalent isotropic displacement parameters (Å^2^)

**Table:**

	*x*	*y*	*z*	*U*_iso_*/*U*_eq_	
O1	0.36588 (15)	0.71186 (11)	0.15883 (12)	0.0579 (5)	
H1	0.3497	0.6666	0.1872	0.087*	
O2	0.50253 (16)	0.63379 (12)	0.31829 (13)	0.0679 (6)	
O3	0.98444 (15)	0.78678 (10)	0.20508 (10)	0.0449 (4)	
O4	0.74288 (15)	1.04026 (10)	0.34010 (10)	0.0474 (4)	
C1	1.01925 (19)	0.76067 (13)	0.28878 (14)	0.0324 (5)	
C2	1.1273 (2)	0.69369 (16)	0.31500 (15)	0.0443 (6)	
H2A	1.2095	0.7257	0.3296	0.053*	
H2B	1.1287	0.6558	0.2597	0.053*	
C3	1.1169 (2)	0.63400 (15)	0.40043 (15)	0.0414 (5)	
C4	1.0993 (2)	0.69384 (14)	0.48413 (14)	0.0365 (5)	
H4A	1.0735	0.6569	0.5333	0.044*	
H4B	1.1821	0.7215	0.5118	0.044*	
C4A	0.99980 (18)	0.76586 (13)	0.45531 (13)	0.0282 (4)	
C5	0.86618 (19)	0.92717 (14)	0.60844 (13)	0.0340 (5)	
H5A	0.9431	0.9626	0.6337	0.041*	
H5B	0.8581	0.8815	0.6556	0.041*	
C5A	0.88328 (18)	0.88242 (13)	0.51737 (13)	0.0281 (4)	
C6	0.74670 (19)	0.98788 (14)	0.59372 (15)	0.0364 (5)	
C7	0.7532 (2)	1.04984 (13)	0.50920 (14)	0.0354 (5)	
H7A	0.6723	1.0833	0.4935	0.043*	
H7B	0.8229	1.0928	0.5294	0.043*	
C8	0.77544 (18)	1.00365 (13)	0.41909 (14)	0.0312 (5)	
C8A	0.83906 (18)	0.91691 (13)	0.42877 (13)	0.0277 (4)	
C9	0.85163 (18)	0.86508 (12)	0.33895 (12)	0.0279 (4)	
H9	0.8768	0.9076	0.2933	0.033*	
C9A	0.95946 (18)	0.79613 (13)	0.36429 (13)	0.0276 (4)	
N10	0.95340 (15)	0.80412 (11)	0.52928 (11)	0.0325 (4)	
H10	0.9686	0.7784	0.5846	0.039*	
C11	1.0003 (3)	0.57076 (16)	0.37230 (17)	0.0570 (7)	
H11A	0.9227	0.6054	0.3505	0.085*	
H11B	0.9906	0.5356	0.4271	0.085*	
H11C	1.0144	0.5316	0.3217	0.085*	
C12	1.2408 (3)	0.5783 (2)	0.43053 (19)	0.0732 (9)	
H12A	1.2543	0.5424	0.3772	0.110*	
H12B	1.2321	0.5399	0.4831	0.110*	
H12C	1.3138	0.6176	0.4502	0.110*	
C13	0.6240 (2)	0.92964 (19)	0.57391 (19)	0.0600 (7)	
H13A	0.5489	0.9666	0.5736	0.090*	
H13B	0.6297	0.8848	0.6231	0.090*	
H13C	0.6163	0.9009	0.5125	0.090*	
C14	0.7475 (3)	1.04393 (17)	0.68425 (17)	0.0587 (7)	
H14A	0.8277	1.0769	0.7002	0.088*	
H14B	0.7398	1.0049	0.7366	0.088*	
H14C	0.6756	1.0851	0.6727	0.088*	
C15	0.72117 (19)	0.82284 (13)	0.29120 (13)	0.0311 (5)	
C16	0.6463 (2)	0.85976 (16)	0.21025 (17)	0.0591 (8)	
H16	0.6756	0.9107	0.1832	0.071*	
C17	0.5277 (3)	0.82234 (17)	0.16818 (18)	0.0640 (8)	
H17	0.4781	0.8491	0.1138	0.077*	
C18	0.4818 (2)	0.74704 (14)	0.20478 (15)	0.0393 (5)	
C19	0.5562 (2)	0.70943 (14)	0.28684 (15)	0.0370 (5)	
C20	0.67392 (19)	0.74706 (14)	0.32934 (14)	0.0362 (5)	
H20	0.7225	0.7211	0.3846	0.043*	
C21	0.5784 (3)	0.58366 (18)	0.3926 (2)	0.0686 (8)	
H21A	0.6595	0.5680	0.3752	0.103*	
H21B	0.5326	0.5299	0.4029	0.103*	
H21C	0.5952	0.6187	0.4505	0.103*	

### Atomic displacement parameters (Å^2^)

**Table:**

	*U*^11^	*U*^22^	*U*^33^	*U*^12^	*U*^13^	*U*^23^
O1	0.0465 (10)	0.0494 (10)	0.0643 (11)	−0.0135 (8)	−0.0210 (8)	0.0109 (8)
O2	0.0477 (10)	0.0733 (12)	0.0732 (12)	−0.0243 (9)	−0.0107 (9)	0.0376 (10)
O3	0.0643 (10)	0.0500 (9)	0.0211 (8)	−0.0007 (8)	0.0103 (7)	−0.0018 (7)
O4	0.0625 (10)	0.0403 (9)	0.0365 (9)	0.0105 (8)	0.0028 (7)	0.0104 (7)
C1	0.0378 (11)	0.0354 (11)	0.0240 (10)	−0.0080 (9)	0.0061 (8)	−0.0028 (9)
C2	0.0458 (13)	0.0560 (14)	0.0338 (12)	0.0059 (11)	0.0144 (10)	−0.0080 (11)
C3	0.0459 (13)	0.0448 (13)	0.0310 (11)	0.0142 (11)	0.0019 (9)	−0.0036 (10)
C4	0.0364 (11)	0.0445 (12)	0.0262 (11)	0.0079 (10)	0.0002 (8)	−0.0001 (9)
C4A	0.0280 (10)	0.0327 (11)	0.0233 (10)	−0.0011 (8)	0.0040 (8)	−0.0022 (8)
C5	0.0342 (11)	0.0421 (12)	0.0245 (10)	0.0040 (9)	0.0032 (8)	−0.0001 (9)
C5A	0.0272 (10)	0.0317 (10)	0.0250 (10)	−0.0007 (8)	0.0043 (8)	0.0000 (8)
C6	0.0327 (11)	0.0422 (12)	0.0352 (11)	0.0048 (10)	0.0091 (9)	0.0032 (9)
C7	0.0324 (11)	0.0332 (11)	0.0404 (12)	0.0022 (9)	0.0069 (9)	0.0003 (9)
C8	0.0262 (10)	0.0319 (11)	0.0333 (11)	−0.0045 (9)	0.0005 (8)	0.0041 (9)
C8A	0.0271 (10)	0.0298 (10)	0.0250 (10)	−0.0025 (8)	0.0026 (8)	0.0015 (8)
C9	0.0328 (10)	0.0291 (10)	0.0203 (9)	−0.0022 (8)	0.0020 (8)	0.0047 (8)
C9A	0.0291 (10)	0.0313 (10)	0.0215 (9)	−0.0037 (8)	0.0027 (7)	−0.0014 (8)
N10	0.0404 (10)	0.0383 (10)	0.0185 (8)	0.0082 (8)	0.0057 (7)	0.0056 (7)
C11	0.0828 (19)	0.0430 (14)	0.0410 (14)	−0.0041 (13)	0.0027 (13)	−0.0003 (11)
C12	0.080 (2)	0.083 (2)	0.0525 (16)	0.0469 (17)	0.0048 (14)	−0.0075 (15)
C13	0.0364 (13)	0.0738 (18)	0.0707 (18)	−0.0037 (13)	0.0128 (12)	0.0242 (14)
C14	0.0721 (18)	0.0621 (16)	0.0480 (15)	0.0236 (14)	0.0269 (13)	0.0006 (12)
C15	0.0354 (11)	0.0303 (11)	0.0244 (10)	−0.0007 (9)	−0.0016 (8)	−0.0002 (8)
C16	0.0675 (17)	0.0463 (14)	0.0490 (15)	−0.0251 (13)	−0.0232 (12)	0.0232 (11)
C17	0.0661 (17)	0.0524 (15)	0.0542 (16)	−0.0174 (13)	−0.0341 (13)	0.0244 (13)
C18	0.0362 (12)	0.0345 (11)	0.0408 (12)	−0.0028 (10)	−0.0072 (9)	−0.0011 (10)
C19	0.0345 (11)	0.0377 (12)	0.0375 (12)	−0.0035 (9)	0.0039 (9)	0.0061 (9)
C20	0.0352 (11)	0.0430 (12)	0.0274 (11)	−0.0003 (10)	−0.0008 (8)	0.0114 (9)
C21	0.0579 (17)	0.0636 (18)	0.083 (2)	−0.0048 (14)	0.0111 (14)	0.0390 (16)

### Geometric parameters (Å, º)

**Table:**

O1—C18	1.364 (2)	C8—C8A	1.448 (3)
O1—H1	0.8200	C8A—C9	1.520 (3)
O2—C19	1.374 (2)	C9—C9A	1.516 (3)
O2—C21	1.403 (3)	C9—C15	1.534 (3)
O3—C1	1.234 (2)	C9—H9	0.9800
O4—C8	1.232 (2)	N10—H10	0.8600
C1—C9A	1.447 (3)	C11—H11A	0.9600
C1—C2	1.499 (3)	C11—H11B	0.9600
C2—C3	1.526 (3)	C11—H11C	0.9600
C2—H2A	0.9700	C12—H12A	0.9600
C2—H2B	0.9700	C12—H12B	0.9600
C3—C4	1.527 (3)	C12—H12C	0.9600
C3—C12	1.528 (3)	C13—H13A	0.9600
C3—C11	1.532 (3)	C13—H13B	0.9600
C4—C4A	1.495 (3)	C13—H13C	0.9600
C4—H4A	0.9700	C14—H14A	0.9600
C4—H4B	0.9700	C14—H14B	0.9600
C4A—C9A	1.354 (2)	C14—H14C	0.9600
C4A—N10	1.368 (2)	C15—C16	1.370 (3)
C5—C5A	1.498 (3)	C15—C20	1.386 (3)
C5—C6	1.525 (3)	C16—C17	1.384 (3)
C5—H5A	0.9700	C16—H16	0.9300
C5—H5B	0.9700	C17—C18	1.364 (3)
C5A—C8A	1.352 (2)	C17—H17	0.9300
C5A—N10	1.371 (2)	C18—C19	1.384 (3)
C6—C7	1.527 (3)	C19—C20	1.379 (3)
C6—C13	1.530 (3)	C20—H20	0.9300
C6—C14	1.532 (3)	C21—H21A	0.9600
C7—C8	1.512 (3)	C21—H21B	0.9600
C7—H7A	0.9700	C21—H21C	0.9600
C7—H7B	0.9700		
			
C18—O1—H1	109.5	C8A—C9—H9	107.9
C19—O2—C21	118.32 (18)	C15—C9—H9	107.9
O3—C1—C9A	120.73 (19)	C4A—C9A—C1	119.19 (18)
O3—C1—C2	120.74 (18)	C4A—C9A—C9	121.81 (17)
C9A—C1—C2	118.50 (17)	C1—C9A—C9	119.00 (16)
C1—C2—C3	114.46 (18)	C4A—N10—C5A	121.63 (16)
C1—C2—H2A	108.6	C4A—N10—H10	119.2
C3—C2—H2A	108.6	C5A—N10—H10	119.2
C1—C2—H2B	108.6	C3—C11—H11A	109.5
C3—C2—H2B	108.6	C3—C11—H11B	109.5
H2A—C2—H2B	107.6	H11A—C11—H11B	109.5
C2—C3—C4	108.56 (18)	C3—C11—H11C	109.5
C2—C3—C12	110.3 (2)	H11A—C11—H11C	109.5
C4—C3—C12	109.17 (17)	H11B—C11—H11C	109.5
C2—C3—C11	109.39 (18)	C3—C12—H12A	109.5
C4—C3—C11	110.29 (19)	C3—C12—H12B	109.5
C12—C3—C11	109.1 (2)	H12A—C12—H12B	109.5
C4A—C4—C3	113.14 (16)	C3—C12—H12C	109.5
C4A—C4—H4A	109.0	H12A—C12—H12C	109.5
C3—C4—H4A	109.0	H12B—C12—H12C	109.5
C4A—C4—H4B	109.0	C6—C13—H13A	109.5
C3—C4—H4B	109.0	C6—C13—H13B	109.5
H4A—C4—H4B	107.8	H13A—C13—H13B	109.5
C9A—C4A—N10	120.24 (17)	C6—C13—H13C	109.5
C9A—C4A—C4	124.55 (17)	H13A—C13—H13C	109.5
N10—C4A—C4	115.15 (16)	H13B—C13—H13C	109.5
C5A—C5—C6	112.54 (16)	C6—C14—H14A	109.5
C5A—C5—H5A	109.1	C6—C14—H14B	109.5
C6—C5—H5A	109.1	H14A—C14—H14B	109.5
C5A—C5—H5B	109.1	C6—C14—H14C	109.5
C6—C5—H5B	109.1	H14A—C14—H14C	109.5
H5A—C5—H5B	107.8	H14B—C14—H14C	109.5
C8A—C5A—N10	120.88 (17)	C16—C15—C20	117.74 (19)
C8A—C5A—C5	123.82 (18)	C16—C15—C9	121.04 (18)
N10—C5A—C5	115.24 (16)	C20—C15—C9	121.21 (16)
C5—C6—C7	107.52 (16)	C15—C16—C17	120.9 (2)
C5—C6—C13	109.03 (18)	C15—C16—H16	119.5
C7—C6—C13	111.54 (18)	C17—C16—H16	119.5
C5—C6—C14	110.03 (17)	C18—C17—C16	121.4 (2)
C7—C6—C14	109.70 (18)	C18—C17—H17	119.3
C13—C6—C14	109.01 (19)	C16—C17—H17	119.3
C8—C7—C6	115.43 (17)	O1—C18—C17	118.83 (19)
C8—C7—H7A	108.4	O1—C18—C19	122.98 (19)
C6—C7—H7A	108.4	C17—C18—C19	118.19 (19)
C8—C7—H7B	108.4	O2—C19—C20	125.45 (18)
C6—C7—H7B	108.4	O2—C19—C18	114.10 (18)
H7A—C7—H7B	107.5	C20—C19—C18	120.45 (19)
O4—C8—C8A	121.28 (18)	C19—C20—C15	121.24 (18)
O4—C8—C7	120.51 (18)	C19—C20—H20	119.4
C8A—C8—C7	118.18 (17)	C15—C20—H20	119.4
C5A—C8A—C8	119.51 (18)	O2—C21—H21A	109.5
C5A—C8A—C9	121.13 (17)	O2—C21—H21B	109.5
C8—C8A—C9	119.33 (16)	H21A—C21—H21B	109.5
C9A—C9—C8A	109.35 (14)	O2—C21—H21C	109.5
C9A—C9—C15	112.51 (15)	H21A—C21—H21C	109.5
C8A—C9—C15	111.07 (15)	H21B—C21—H21C	109.5
C9A—C9—H9	107.9		
			
O3—C1—C2—C3	−150.88 (19)	N10—C4A—C9A—C9	−7.0 (3)
C9A—C1—C2—C3	31.0 (3)	C4—C4A—C9A—C9	175.87 (18)
C1—C2—C3—C4	−52.0 (2)	O3—C1—C9A—C4A	179.86 (19)
C1—C2—C3—C12	−171.60 (19)	C2—C1—C9A—C4A	−2.1 (3)
C1—C2—C3—C11	68.3 (2)	O3—C1—C9A—C9	0.9 (3)
C2—C3—C4—C4A	46.2 (2)	C2—C1—C9A—C9	178.97 (17)
C12—C3—C4—C4A	166.4 (2)	C8A—C9—C9A—C4A	21.9 (2)
C11—C3—C4—C4A	−73.7 (2)	C15—C9—C9A—C4A	−102.0 (2)
C3—C4—C4A—C9A	−20.7 (3)	C8A—C9—C9A—C1	−159.14 (16)
C3—C4—C4A—N10	162.08 (18)	C15—C9—C9A—C1	76.9 (2)
C6—C5—C5A—C8A	26.8 (3)	C9A—C4A—N10—C5A	−11.1 (3)
C6—C5—C5A—N10	−156.02 (17)	C4—C4A—N10—C5A	166.29 (17)
C5A—C5—C6—C7	−50.7 (2)	C8A—C5A—N10—C4A	11.0 (3)
C5A—C5—C6—C13	70.3 (2)	C5—C5A—N10—C4A	−166.26 (17)
C5A—C5—C6—C14	−170.15 (18)	C9A—C9—C15—C16	−133.8 (2)
C5—C6—C7—C8	50.7 (2)	C8A—C9—C15—C16	103.3 (2)
C13—C6—C7—C8	−68.8 (2)	C9A—C9—C15—C20	47.4 (2)
C14—C6—C7—C8	170.34 (18)	C8A—C9—C15—C20	−75.6 (2)
C6—C7—C8—O4	157.24 (18)	C20—C15—C16—C17	−0.1 (4)
C6—C7—C8—C8A	−24.5 (2)	C9—C15—C16—C17	−179.0 (2)
N10—C5A—C8A—C8	−174.53 (17)	C15—C16—C17—C18	−0.9 (5)
C5—C5A—C8A—C8	2.5 (3)	C16—C17—C18—O1	−178.3 (3)
N10—C5A—C8A—C9	7.2 (3)	C16—C17—C18—C19	1.2 (4)
C5—C5A—C8A—C9	−175.83 (17)	C21—O2—C19—C20	−8.0 (4)
O4—C8—C8A—C5A	174.37 (19)	C21—O2—C19—C18	171.2 (2)
C7—C8—C8A—C5A	−3.9 (3)	O1—C18—C19—O2	−0.3 (3)
O4—C8—C8A—C9	−7.3 (3)	C17—C18—C19—O2	−179.9 (2)
C7—C8—C8A—C9	174.46 (17)	O1—C18—C19—C20	178.8 (2)
C5A—C8A—C9—C9A	−21.9 (2)	C17—C18—C19—C20	−0.7 (4)
C8—C8A—C9—C9A	159.82 (16)	O2—C19—C20—C15	178.8 (2)
C5A—C8A—C9—C15	102.9 (2)	C18—C19—C20—C15	−0.3 (3)
C8—C8A—C9—C15	−75.4 (2)	C16—C15—C20—C19	0.6 (3)
N10—C4A—C9A—C1	174.02 (17)	C9—C15—C20—C19	179.51 (19)
C4—C4A—C9A—C1	−3.1 (3)		

### Hydrogen-bond geometry (Å, º)

**Table:**

*D*—H···*A*	*D*—H	H···*A*	*D*···*A*	*D*—H···*A*
O1—H1···O4^i^	0.82	2.12	2.800 (2)	141
N10—H10···O3^ii^	0.86	1.95	2.802 (2)	174

Symmetry codes: (i) −*x*+1, *y*−1/2, −*z*+1/2; (ii) *x*, −*y*+3/2, *z*+1/2.
